# Cervical Cancer Screening and Associated Factors Among Women of Reproductive Age in Sidama Regional State of Ethiopia: A Cross‐Sectional Survey

**DOI:** 10.1002/cnr2.70498

**Published:** 2026-02-16

**Authors:** Getu Mune, Amare Asefa, Serawit Lakew Chillo, Endrias Markos Woldesemayat

**Affiliations:** ^1^ South Ethiopia Regional Health Burao, Gedeo Zone Health Office Dilla Ethiopia; ^2^ Department of Oncology College of Medicine and Health Sciences, Jimma University Jimma Ethiopia; ^3^ School of Nursing and Midwifery College of Medicine and Health Sciences, Arba Minch University Arba Minch Ethiopia; ^4^ School of Public Health College of Medicine and Health Sciences, Hawassa University Hawassa Ethiopia

**Keywords:** attitude, cervical cancer screening, Ethiopia, knowledge, practice

## Abstract

**Background:**

Africa and sub‐Saharan Africa reported higher cervical cancer cases globally. Cervical cancer is the second most leading cause of cancer in Ethiopia. Yet, the evidence was low for cervical cancer screening practices in Ethiopia.

**Aim:**

In this study, we assessed the cervical cancer screening and associated factors among reproductive‐age women in Sidama Region, Bensa, Ethiopia.

**Methods:**

A facility‐based cross‐sectional study design was conducted from January 1, 2023 to March 30, 2023 in Bensa district. Data were collected using a pre‐tested and structured questionnaire. Multivariable logistic regression was used to identify the associated factors with knowledge, attitude, and practice of cervical cancer screening.

**Results:**

One hundred forty‐seven 147 (36.7%) had good knowledge, 218 (54.4%) had a favorable attitude towards cervical cancer screening and only 60 (15%) respondents were screened for cervical cancer. Women having better educational status was 1.6 (AOR: 95% CI: 1.0, 2.5) times more likely to have knowledge of cervical cancer screening. Women being the protestant religion followers (AOR: 1.9; 95% CI; 1.0–3.4), Sidama ethnic group (AOR: 4.5; 95% CI: 2.1–9.7), having no formal education (AOR: 1.7; 95% CI: 1.1–2.7), and having good knowledge of cervical cancer (AOR: 2.2; 95% CI: 1.4–3.4) were associated with a positive attitude towards screening for cervical cancer. The odds of cervical cancer screening were low among Amhara's ethnic categories (AOR: 0.2; 95% CI: 0.1–0.5), while higher in women with single marital statuses (AOR: 2.4; 95% CI: 1.2–4.8), and those who have information about cancer screening (AOR: 2.0; 95% CI: 1.1–3.8).

**Conclusion:**

The results of this study showed that cervical cancer screening practice was low. The respondents' knowledge and education status were positively associated with screening for cervical cancer.

## 1 Introduction

Cervical cancer is a significant public health issue worldwide and remains one of the leading causes of cancer‐related deaths among women, particularly in developing countries [1]. The primary cause of cervical cancer is persistent infection with the human papillomavirus (HPV) transmitted majorly by unprotected sexual intercourse [2].

Globally, cervical cancer is the 4th leading cause of female cancer and the 2nd causes of female cancer in women aged 15–44 years, with an estimated 570 000 new cases and 311 000 deaths reported annually [3]. Developing countries account for more than 85% of these cases, mainly due to limited access to screening programs, inadequate healthcare infrastructure, and low awareness levels [4]. Sub‐Saharan Africa is the most affected countries with cervical cancer in the world in which nearly100% of the cases are related to human papilloma virus [5].

According to the World Health Organization (WHO), the global strategy to accelerate the elimination of cervical cancer requires countries to adopt a three‐pillar approach. This strategy sets targets of 90% for vaccination, 70% for screening, and 90% for treatment by 2030 [6]. Despite cervical cancer screening has been proven to reduce the incidence of cervical cancer, various studies in developing countries show that the uptake of cervical cancer screening is highly inadequate [4]. This inadequacy is due to a lack of awareness about cervical cancer, attitude of women toward cervical cancer screening, very low coverage of screening services, poor health‐seeking behavior, a weak health system, insufficient healthcare resources to sustain screening programs, and various socio‐cultural factors [4, 7].

As the WHO suggests that globally 30% of the burden related to all cancers can be reduced with early detection and treatment [8]. Cervical cancer screening can be early diagnosed using detecting service technologies such as PaP smear test, VIA, HPV DNA, and liquid based cytology LBC [8].

The overall cervical cancer screening practice in Africa is 20.9%, with a 95% CI of 15.84%–26.04% [9]. In Kenya, the cervical cancer screening program was poor and inadequate. Evidence‐based policy implementation and sustained health system strengthening are necessary to move towards cervical cancer elimination as a public health problem [10].

In Ethiopia, 36.9 million women aged 15 and older are at risk of developing cervical cancer. Annually, an estimated 7445 women are diagnosed with cervical cancer, and 5338 die from the disease [11]. The vulnerability is related to low HPV vaccination coverage, early initiation of sexual intercourse, early marriage [12], and limited knowledge about sexual and reproductive health issues [13]. Therefore, understanding the knowledge and attitudes of reproductive‐aged women regarding cervical cancer and their screening practices is crucial for developing an effective implementation program. Little is known about the specific knowledge, attitudes, and practices of women in Bensa District, where cultural and structural barriers could have further influenced the screening behaviors. Assessing these factors is therefore critical to inform targeted interventions, strengthen local screening programs, and align with the World Health Organization's global strategy to eliminate cervical cancer through vaccination, screening, and treatment [14]. Therefore, this study aimed to assess the level of cervical cancer screening practices and associated factors among reproductive‐aged women at public health facilities in the Bensa District, Sidama Region, Ethiopia.

## 2 Materials and Methods

### 2.1 Study Design and Setting

A facility‐based cross‐sectional study design was conducted from January 1, 2023 to March 30, 2023 at Bensa District, of Sidama Region in Ethiopia. Bensa District is located 400 km from Addis Ababa, the capital of Ethiopia, and 131 km from Hawassa, the capital of the Sidama Region. The district has an average altitude of 1914 m above sea level and receives an average annual rainfall of 1251.2 mm. According to the Bensa District Health Office report of 2022, the district has a total population of 203 792, with 99 653 males and 104 136 females. Bensa District comprises 24 kebeles, 22 of which are rural and 2 are urban. The district is served by one primary hospital and seven health centers. Currently, all public health centers provide cervical cancer screening and first treatment services.

### 2.2 Population

The source population comprised all women aged 15–49 years who were obtaining services from health facilities in the Bensa District. The study population was systematically selected women of reproductive age group who were attending selected health facilities in Bensa District during the study period.

### 2.3 Eligibility Criteria

All reproductive age women who attended EPI, ANC, OPD, family planning, and post‐natal care services at the selected health facilities and who had sexual exposure were included in the study. Whereas we excluded women who were critically (severely) ill and those who had resided in Bensa District for less than six months. The exclusion of critically ill participants was because they were incapable of being interviewed.

### 2.4 Sample Size Determination

The sample size was calculated using a single population proportion formula based on a knowledge of cervical cancer screening of 43.1% (0.431), from the study conducted in Wolaita [15]. With a 95% confidence interval and 5% margin of error, the calculated sample size was 377. After adding a 10% non‐response rate, the final sample size was 415.

### 2.5 Sampling Procedure

In Bensa District, there are eight public health facilities offering a comprehensive range of services including EPI, ANC, delivery, family planning, and OPD services. From these facilities, we selected three health centers: Allo Health Center, Oda Health Center, and Hatessa Health Center using simple random sampling. Upon reviewing the client registration log books, the average client flow of women in the reproductive age group was 2621 at Allo Health Center, 6121 at Oda Health Center, and 5292 at Hatessa Health Center. Based on these client flows, the sample size was allocated proportionally to each health center. Finally, we employed systematic random sampling to select the study participants.

### 2.6 Data Collection Tool and Procedure

Data were collected from each study participant using a pre‐tested, structured, interviewer‐administered questionnaire. The questionnaire was developed after reviewing various literatures [15, 16, 17, 18]. The questionnaire consisted of four sections: sociodemographic characteristics, knowledge, attitude, and practices related to cervical cancer screening among the participants. The reliability (Cronbach's alpha: 0.77) of the questionnaire was checked during the pretest period based on unidimensionality assumption for scale response. Questionnaire was initially prepared in English and then translated into the local language Sidamu Afoo local language, and back to English to ensure consistency. Data were collected by three BSc nurses in the health centers and supervised by two BSc public health officers.

### 2.7 Data Quality Control

Data collectors received 2 days of training on the study's purpose, the data collection tool, and the study's ethical aspects. A pre‐test was conducted on 5% of the total sample size with patients at Worancha Health Center in Bona Woreda before the actual study to validate the questionnaire's content. Then corrective measures such as changing the sequencing of the questions and changing some wording were done. The principal investigator provided daily on‐site supervision throughout the data collection period. At the end of each day, the completeness of the information was audited to ensure accuracy.

### 2.8 Variables of the Study

Dependent variables considered in the study were Knowledge, Attitude and Practice of Cervical Cancer screening. Independent variables that were measured in the study were Socio‐demographic Characteristics (Age, Marital status, educational status, Religion, Income, Occupation, sources of information). Lifestyle and behavioral factors (Early initiation of sex, multiple sexual partners, History of STIs).

### 2.9 Operational Definitions

#### 2.9.1 Knowledge About Cervical Cancer Screening

We used 11 questions to assess the study subjects' knowledge level about cervical cancer screening. Those who scored at or above the mean value were classified as having good knowledge, while those who scored below the mean value were classified as having poor knowledge [16].

#### 2.9.2 Attitude

Attitude was assessed using 7 Likert scale questions. Responses were coded as follows: Strongly disagree = 1, Disagree = 2, Neutral = 3, Agree = 4, and Strongly agree = 5. The responses were summed to obtain a total score for each respondent. Those with scores above the mean were labeled as having a positive attitude toward cervical cancer screening, while those with scores below the mean were considered to have a negative attitude [16].

#### 2.9.3 Practice Cervical Cancer Screening

Respondents who had undergone cervical cancer screening at least once were considered to have practiced cervical cancer screening. However, respondents who had never undergone the procedure were considered not to have practiced cervical cancer screening [16].

### 2.10 Data Processing and Analysis

Data entry was done using EPI‐INFO version 7.2.1.0, and exported to SPSS version 26 for analysis. Descriptive analysis was performed to present the dependent and independent variables using frequency, percentile, and the mean. Binary logistic regression analysis was used to identify the factors associated with the outcome variable. In the bivariate analysis, those variables with *p* < 0.25 were considered as candidates for the multivariate analysis. More over multiconallnearity was checked by using collinearity diagnostic test by checking the value of variance inflation factor and tolerance test. Hosmer‐lemeshow goodness of fit was used to test for the model fitness. Odds ratio at 95% CI were used to measure strength of association between outcome and predictor variables. *p* < 0.05 was considered to declare statistically significant in multivariate logistic regression analysis. The results were presented in texts, figures, and tables. Missing data was handled using complete case analysis approach.

## 3 Results

### 3.1 Socio‐Demographic Characteristic of Respondents

Out of 415 participants, 401 were included in the study, with a response rate of 96.6%. Majority of the respondents 335 (83.5%) reported that they are sexually active. Among those who started sexual intercourse, 322 (80.3%) had single sexual partner, and 13 (3.2%) had multiple sexual partners. More than half 222 (55.4%) of the study participants were from urban areas. Concerning educational status, 261 (65.1%) had no formal education (Table 1).

**TABLE 1:** Socio‐demographic and Sexual experience related characteristics of women attending at health facilities in Bensa District, Sidama Region, Ethiopia (*n* = 415).

Variable	Categories	Frequency (%)
Age	20_39	290 (72.3)
	> 40	111 (27.7)
Marital status	Single	66 (16.5)
	Married	335 (83.5)
Religion	Protestant	334 (83.3)
	Orthodox	42 (10.5)
	Muslim	25 (6.2)
Ethnicity	Sidama	235 (58.6)
	Amhara	128 (31.9)
	Wolaita	38 (9.5)
Place of residence	Urban	222 (55.4)
	Rural	179 (44.6)
Educational status	Informal education	261 (65.1)
	Primary	140 (34.9)
Sexual experience	Yes	335 (83.5)
	No	66 (16.5)
Sexual partner	Single	322 (80.3)
	Multiple	13 (3.2)
	No formal sexual partner	66 (16.5)

### 3.2 Cervical Cancer Screening Related Knowledge, Attitude and Practice of Respondents

More than one third, 147 (36.7%; 95% CI: 30.9–41.4) of participants had good knowledge about cervical cancer screening. More than half of the participants, 210 (52.4%) had heard about cervical cancer, with the media being the primary source of information 101 (48.1%). Only 110 (27.4%), 81 (20.2%), and 68 (16.9%) identified vaginal bleeding, foul‐smelling vaginal discharge, and pain during sexual intercourse as symptoms of cervical cancer, respectively, while the remaining 35.5% participants were unaware of the symptoms. Regarding treatment, 55 (13.7%) participants said cervical cancer can be treated if identified early, while the remaining 81 (20.2%) think it is not treatable, and 265 (66.1%) were not sure. Regarding the recommended frequency of cervical cancer screening, 10 participants (2.5%) said annually,31 (7.7%) participants said every 3 years, 157 (39.2%) participants said every 5 years, and 203 participants (50.6%) did not provide a response (Table 2).

**TABLE 2:** Knowledge, attitude and practice of cervical cancer screening among reproductive age group women attending at health facilities in Bensa District, Sidama Region, Ethiopia (*n* = 415).

Variables	Category	Number	Percent (%)
Information of cervical cancer	Yes	210	52.4
	No	191	47.6
Source of information	News media	101	48.1
	Health workers	90	42.9
	Family or friends	19	9.0
Risk factors for cervical cancer	Multiple sexual partner	385	96.0
	Early sexual intercourse	11	2.7
	Other	5	1.2
Cervical cancer symptoms	Vaginal bleeding	110	27.4
	Vaginal foul‐smelling discharge	81	20.2
	Pain during sex	68	16.9
	Don't know	142	35.5
How can cervical cancer be prevented?	Avoid multiple sexual partner	305	76.1
	Avoid early sexual intercourse	94	23.4
	others	2	0.5
Cervical cancer can be treated	Yes	55	13.7
	No	81	20.2
	I don't know	265	66.1
Treatment of cervical cancer	herbal remedies	32	8.0
	surgery	139	34.7
	by drug	194	48.4
	don't know	36	9.0
Know cost of cervical cancer treatment	free	83	20.7
	very expensive	103	25.7
	moderate expensive	155	38.7
	don't know	60	15.0
Frequency of screening	once every year	10	2.5
	once every 3 years	31	7.7
	once every 5 years	157	39.2
	others	203	50.6
Who should be screened?	Women of > 25 years	58	14.5
	Prostitutes	182	45.4
	Others	161	40.1
Screening procedures	Visual inspection with acetic acid	19	4.7
	Pap smear	31	7.7
	Biopsy	45	11.2
	Don't know	306	76.3
Knowledge	Poor	254	63.3
	Good	147	36.7
Attitude	Negative	183	45.6
	Positive	218	54.4
Practice	No	341	85.0
	Yes	60	15.0
Where have you been screened	Hospital	12	20.0
	Health center	48	80.0
Reasons for not screened	Not knowing where to get the service	75	22.0
	Absence of symptoms	196	54.6
	Screening was too expensive	38	11.1
	Negative attitude toward screening	42	12.3

More than half of the respondents, 218 (54.4%; 95% CI: 49.6–59.1), had positive attitude toward cervical cancer screening. Three hundred eighteen respondents (79.3%) perceived that any woman can acquire cervical cancer. Two hundred ninety‐six respondents (73.8%) agreed that screening helps to prevent cervical cancer, and 332 respondents (82.8%) stated they would volunteer for screening if it were free. Only 330 respondents (82.3%) believed they were at risk of acquiring cervical cancer.

Most of the respondents 341 (85%) had not been screened for cervical cancer, with only 15% (95% CI: 11.5, 18.5) having been screened at some point in their lives. When asked for reasons for not being screened, 186 (54.6%) respondents said it was because there had no any symptoms, and 75 (22.0%) said they didn't know where to get the service (Table 2).

### 3.3 Factors Associated With Knowledge of Cervical Cancer Screening

Women who are living in urban areas were 2 times more likely to have good knowledge about cervical cancer screening when compared to women living in rural areas (AOR: 95% CI: 1.12, 3.10). Additionally, women who have completed primary education or higher were 1.6 times more likely to have good knowledge about cervical cancer screening compared to no formal education (AOR: 95% CI: 1.0, 2.5) (Table 3).

**TABLE 3:** Factors associated with knowledge of cervical cancer screening among reproductive age group women attending health facilities in Bensa District, Sidama Region, Ethiopia (*n* = 415).

Variables	Knowledge of screening		COR (95% CI)	AOR (95% CI)
	Good	Poor		
Ethnicity				
Sidama	83	152	0.5 (0.3–0.9)	0.5 (0.3–1.0)
Amhara	44	84	0.5 (0.2–0.9)	0.5 (0.2–1.1)
Wolaita	20	18		
Marital status				
Married	118	217	1.4 (0.8–2.5)	1.4 (0.8–2.4)
Single	29	37	1	1
Place of residence				
Rural	59	120	0.7 (0.5–1.1)	0.8 (0.6–1.3)
Urban	88	134	1	1
Educational status				
No formal education	84	177	1	1
At least primary	63	77	1.7 (1.1–2.6)	1.6 (1.0–2.5)

### 3.4 Factors Associated With Attitude Towards Cervical Cancer Screening

The protestant religion follower women were (AOR: 1.9; 95% CI: 1.0–3.4) times more likely to have a positive attitude towards cervical cancer screening compared to women in other religions. Sidama women were more likely to have a positive attitude towards cervical cancer screening than women in other ethnic groups (AOR: 4.5; 95% CI: 2.1–9.7). Women's educational status having no formal education had good attitudes compared to those who achieved at least primary (AOR: 1.7; 95% CI: 1.1–2.7). Women with good knowledge had increased odds of good attitude (AOR: 2.2; 95% CI: 1.4–3.4) (Table 4).

**TABLE 4:** Factors associated with the attitude toward cervical cancer screening among reproductive age women attending at health facilities in Bensa District, Sidama Region, Ethiopia (*n* = 415).

Variables	Attitude towards cervical cancer screening		COR (95% CI)	AOR (95% CI)
	Positive	Negative		
Religion				
Protestant	172	162	2.0 (1.2–3.6)	1.9 (1.0–3.4)
Others	46	21		1
Ethnicity				
Sidama	147	88	4.1 (1.9–8.7)	4.5 (2.1–9.7)
Amhara	60	68	2.1 (0.9–4.7)	2.2 (0.9–4.8)
Wolaita	11	27		1
Educational status				
No formal education	153	108	1.6 (1.1–2.5)	1.7 (1.1–2.7)
At least primary	65	75		1
Knowledge				
Poor	125	129	1	1
Good	93	54	1.8 (1.2–2.7)	2.2 (1.4–3.4)

### 3.5 Factors Associated With the Practice of Cervical Cancer Screening

The odds of cervical cancer screening were lower among Amhara ethnic groups (AOR: 0.2; 95% CI: 0.1–0.5). Single women had over 2.4 times increased odds of cervical cancer screening (AOR: 95% CI: 1.2–4.8). Moreover, women who had information about cervical cancer screening were 2 times more likely to practice cervical cancer screening when compared to their counterparts (AOR: 95% CI: 1.1–3.8) (Table 5).

**TABLE 5:** Factors associated with practice of cervical cancer screening among reproductive age women attending health facilities in Bensa District, Sidama Region, Ethiopia (*n* = 415).

Variables	Practice		COR (95% CI)	AOR (95% CI)
	Yes	No		
Ethinicity				
Sidama	41	194	0.7 (0.3–1.5)	0.5 (0.2–1.2)
Amhara	10	118	0.3 (0.1–0.7)	0.2 (0.1–0.5)
Wolayta	9	29		1
Marital status				
Single	15	51	1.9 (0.9–3.7)	2.4 (1.2–4.8)
Married	45	290		1
Education				
No formal education	49	212	1.16 (0.62–2.18)	1.25 (0.65–2.4)
At least primary	11	129		1
Number of sexual partner				
Single	46	276	0.9 (0.4–1.9)	0.9 (0.4–2.2)
No partner	4	9	2.5 (0.6–9.7)	3.4 (0.8–14.3)
Multiple	10	56		1
Have information about cervical cancer				
No	24	167		1
Yes	36	174	1.4 (0.8–2.5)	2.0 (1.1–3.8)

## 4 Discussion

This study assessed the knowledge, attitudes, and practices regarding cervical cancer screening among women of reproductive age in the Bensa District of the Sidama region, South Ethiopia. The findings revealed that a low proportion of participants had good knowledge, a positive attitude, and were practicing cervical cancer screening. Educational status emerged as the primary factor significantly linked to having good knowledge of cervical cancer screening. Being a Protestant religion follower, Sidama ethnic group, low educational status, and possessing good knowledge were factors that increased the likelihood of a positive attitude toward cervical cancer screening. Factors such as ethnicity, marital status, and access to information about cervical cancer screening were associated with the actual practice of cervical cancer screening.

The level of knowledge regarding screening for cervical cancer in the study district was low. This finding is comparable to studies in Tanzania [19], and India (Perambalur district) [20]. However, it is lower than studies conducted in Ethiopia (Wolaita Zone) (36% vs. 46.1%) [15, 21] and Dessie town, Northeast Ethiopia (36% vs. 51%) [21]. The status of variation in community exposure to social media could be a possible reason for the variation of knowledge about the screening practices.

According to this study, women residing in urban areas were more likely to have good knowledge about cervical cancer screening compared to their rural counterparts. This finding is supported by study conducted in Assosa zone, Ethiopia [22]. This could be explained by the fact that, women in urban areas typically have better access to health information through various channels, including mass media, internet, and health campaigns.

Better education status had association with knowledge of cervical cancer screening. This was in line with former study report from Debre Tabor Town [23], Wolaita Zone [15], and Adigrat Town of Ethiopia [24]. The finding shows that education is a weapon to transmit knowledge to participants. This is because, education improves a person's ability to understand health information, and educated women are more likely to access and utilize various sources of health information, including the internet, books, and educational programs. To bridge the knowledge gap between educated and illiterate women, it is crucial to develop and deliver educational programs tailored to women with low literacy levels.

More than half of participants attitude towards the screening for cervical cancer was favorable. This is in line with the study report from Adigrat town (53.3%) [16], and Gondar (58.2%), North West Ethiopia [25]. However, it is notably lower than study conducted in Zaria, Nigeria (80.4%) [26]. Several factors could contribute to this discrepancy, such as cultural beliefs and norms. In some regions, traditional beliefs and norms about privacy might discourage women from undergoing cervical cancer screening, as the procedure involves showing private parts. This cultural barrier might be more pronounced in the current study district as compared to west Africa (Nigeria).

In this study, a few participants had ever practiced cervical cancer screening. This was consistent with overall practice in Africa (15% vs. 20.9%) [9] and sub‐Saharan Africa (15% vs. 19%) [27], Durame town of central Ethiopia (15% vs. 13.8%) [28], and Butajira town of central Ethiopia (15% vs. 15.1%) [29]. However, it is lower than the report from Addis Ababa, Ethiopia (15% vs. 25%) [18] and higher than a recent report in Kisumu Municipality of rural Kenya (15% Vs. 6%) [30]. The discrepancy here and there could be related to variation in status of knowledge, attitude, level of education, and residency in urban area.

Having information about cervical cancer and educational status were factors that showed association with the practice of cervical cancer screening. This is an implication that information is a milestone for health care practices as shown elsewhere [24].

Sociocultural factors, such as ethnic status and marital status, were among the others having association with cervical cancer screening. We suggest that the educational interventions should take into consideration the sociocultural factors while health information education and communication (IEC) of women takes place in Ethiopia for cervical cancer screening practices.

### 4.1 Implications

As findings suggested, educational empowerment of women could benefit the long‐term success of cervical cancer screening in the study region.

## 5 Limitations of the Study

### 5.1 Strengths

Data quality could have been maintained through tool validation during the pretest period.

#### 5.1.1 Limitations

Since it relied on self‐reported data from the study participants, there may be a risk of social desirability bias. Due to the cross‐sectional nature of the study's design, establishing cause‐and‐effect relationships between variables becomes affected. Analytical study designs such as cohort study designs or case control study designs are appropriate to evaluate such relationships. Lastly, since this study was conducted at health facilities, the generalizability of findings could be limited to the study district. Selection bias and recall bias were another that should be given emphasis. The small outcome frequencies could potentially cause overfitting bias in the results and dichotomizing the scale could have caused oversimplification of complex behaviors.

## 6 Conclusions and Recommendations

The results of this study showed that knowledge and attitudes toward cervical cancer screening were moderate, but the actual screening practices were low. Therefore, it is essential to enhance women's knowledge and attitudes towards cervical cancer and its screening services by developing targeted interventions, particularly in areas with low educational levels, rural areas, and among low‐income women. Additionally, it is crucial to train health professionals to provide their patients comprehensive information and counseling about cervical cancer screening and to actively communicate the benefits and procedures of screening to their patients. Clinically, the results could be a lesson to oncology centers to scale up the screening program to community level.

## Author Contributions

**Getu Mune:** data curation, investigation, conceptualization, formal analysis, writing original draft and review and editing. **Amare Asefa:** conceptualization, investigation, formal analysis, writing original draft, review and editing. **Serawit Lakew Chillo:** conceptualization, investigation, formal analysis, writing original draft, review and editing. **Endrias Markos Woldesemayat:** conceptualization, investigation, formal analysis, writing original draft, review and editing. All authors reviewed the manuscript.

## Funding

The authors have nothing to report.

## Ethics Statement

Ethical clearance was obtained from the Institutional Review Board of Hawassa University, under approval number IRB/101/14 at December 22, 2022. Hawassa University is authorized to give research ethics approval letter in south Ethiopia. Adult hood in Ethiopia begins at the age of 18 years [31]. We have obtained consent from each participant. Minors (< 18 years) were consented from parents or legal guardians [32]. However, the Ethiopian national research ethics guideline allows consent without parental consent for emancipated minors in Ethiopia [32]. The IRB has approved this consent procedure. Permission was also obtained from Bensa woreda Health office and a formal letter was submitted to the selected health centers. After discussing the study's objectives, risks, and benefits, volunteer written consent was obtained from each study subject. Personal identities were removed from the questionnaires, and the information was kept private. Participants were given assurance that they might withdraw or refuse to participate in the study without fear of being judged.

## Conflicts of Interest

The authors declare no conflicts of interest.

## Data Availability

The data that support the findings of this study are available from the corresponding author upon reasonable request.
